# Role of CPEBs in Learning and Memory

**DOI:** 10.1111/jnc.70226

**Published:** 2025-09-08

**Authors:** David A. Hicks, Liam D. Aubrey, Jessica C. F. Kwok, Sheena E. Radford

**Affiliations:** ^1^ Astbury Centre for Structural Molecular Biology, School of Molecular and Cellular Biology, Faculty of Biological Sciences University of Leeds Leeds UK; ^2^ School of Biomedical Sciences, Faculty of Biological Sciences University of Leeds Leeds UK

**Keywords:** AMPA, amyloid, CPEB, memory, neuroscience, RNA‐binding protein

## Abstract

Memory formation involves a complex interplay of molecular and cellular processes, including synaptic plasticity mechanisms such as long‐term potentiation (LTP) and long‐term depression (LTD). These processes rely on activity‐dependent gene expression and local protein synthesis at synapses. A central unresolved question in neuroscience is how memories can be stably maintained over time, despite the transient nature of the proteins involved in their initial encoding. A key candidate addressing this ‘maintenance paradox’ is the CPEB (cytoplasmic polyadenylation element‐binding protein) family, particularly CPEB3. CPEBs are RNA‐binding proteins that regulate the polyadenylation and translation of dormant mRNAs, enabling synaptic tagging and memory consolidation. CPEB3 has been shown to modulate the expression of critical synaptic proteins, including AMPA and NMDA receptor subunits, thereby influencing synaptic strength and long‐term memory persistence. Structurally, CPEB3 features a disordered N‐terminal domain (NTD) enriched in glutamine and proline residues, which may facilitate reversible aggregation and phase separation and an actin‐binding domain, potentially supporting its localisation to ribonucleoprotein granules. The highly conserved C‐terminal domain (CTD) contains RNA‐recognition motifs essential for mRNA binding. Together, these structural features may enable CPEB3 to function as a molecular switch, linking synaptic activity to enduring changes in protein synthesis and memory encoding. Here, we review the current understanding of the function of CPEB3, highlighting current hypotheses and debates of the role(s) of protein self‐assembly in memory formation.

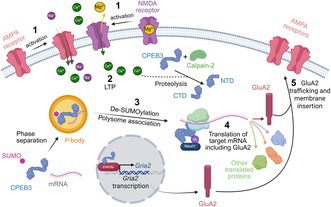

## Graphical abstract

SUMOylation of CPEB3 and its association with target mRNA can lead to phase separation and localisation to P bodies, where translation of target mRNAs is repressed. (1) Activation of AMPA and NMDA receptors leads to (2) long‐term potentiation (LTP). Increased cellular Ca^2+^ concentration leads to activation of calpain‐2 and some proteolysis of CPEB3. (3) LTP leads to deSUMOylation of CPEB3 and translocation out of P bodies. (4) Subsequent oligomerisation of CPEB3 and its mono‐ubiquitination by Neurl1 leads to CPEB3 association with polysomes and translation of target mRNAs, including GluA2. (5) This results in increased AMPA subunit trafficking to the cell surface and membrane insertion of functional AMPA receptors. In addition, CPEB3 is able to modulate GluA2 indirectly through STAT5b.

**Abbreviations**
AAR: amino acid repeat
AD: Alzheimer's disease
AMPA: α‐amino‐3‐hydroxy‐5‐methyl‐4‐isoxazolepropionic acid
CaMKII: Ca^2+^/calmodulin‐dependent protein kinase II
CPEB: cytosolic polyadenylation element‐binding protein
DSCAM: Down's syndrome cell adhesion molecule
FMRP: Fragile X mental retardation protein
FXS: Fragile X syndrome
IMM: intermediate mesopallium
LCD: low‐complexity domain
LTD: long‐term depression
LTP: long‐term potentiation
mPFC: medial prefrontal cortex
NMDA: *N*‐methyl‐d‐aspartate
PPII: polyproline II
PrLD: prion‐like domain
RBP: RNA‐binding protein
RNP: ribonucleoprotein
RRM: RNA‐recognition motif
SFPQ: splicing factor proline–glutamine‐rich

## 1 Introduction

In essence, memory is the system by which daily experiences are recorded and retained, enabling acquisition of knowledge. It operates through four stages: encoding, consolidation, storage and recall (de Ortega‐ San Luis and Ryan 2022; Merlo et al. 2024). Consolidation of memories is predicated on synaptic modifications and the strengthening of neuronal connections within specific brain regions. Neuronal ensembles that are activated both during learning and retrieval processes are known as engram cells, which reside, *inter alia*, in the hippocampus, amygdala and medial prefrontal cortex (mPFC). However, the molecular mechanisms that connect memory acquisition to its subsequent retrieval remain incompletely understood (Kupke and Oliveira 2025). In this review, we discuss knowledge of the formation and retrieval of memories, alongside current knowledge of how memories may be made and retrieved at a molecular level, focusing on the CPEB family of proteins thought to be key players in these processes (Huang et al. 2023). We point out key questions and current hypotheses and show how, by linking the fields of neuroscience and structural biology, we are beginning to gain a foothold into understanding this fundamental feature of human biology.

### 1.1 Biology of Long‐Term and Short‐Term Memory

**FIGURE 1:**
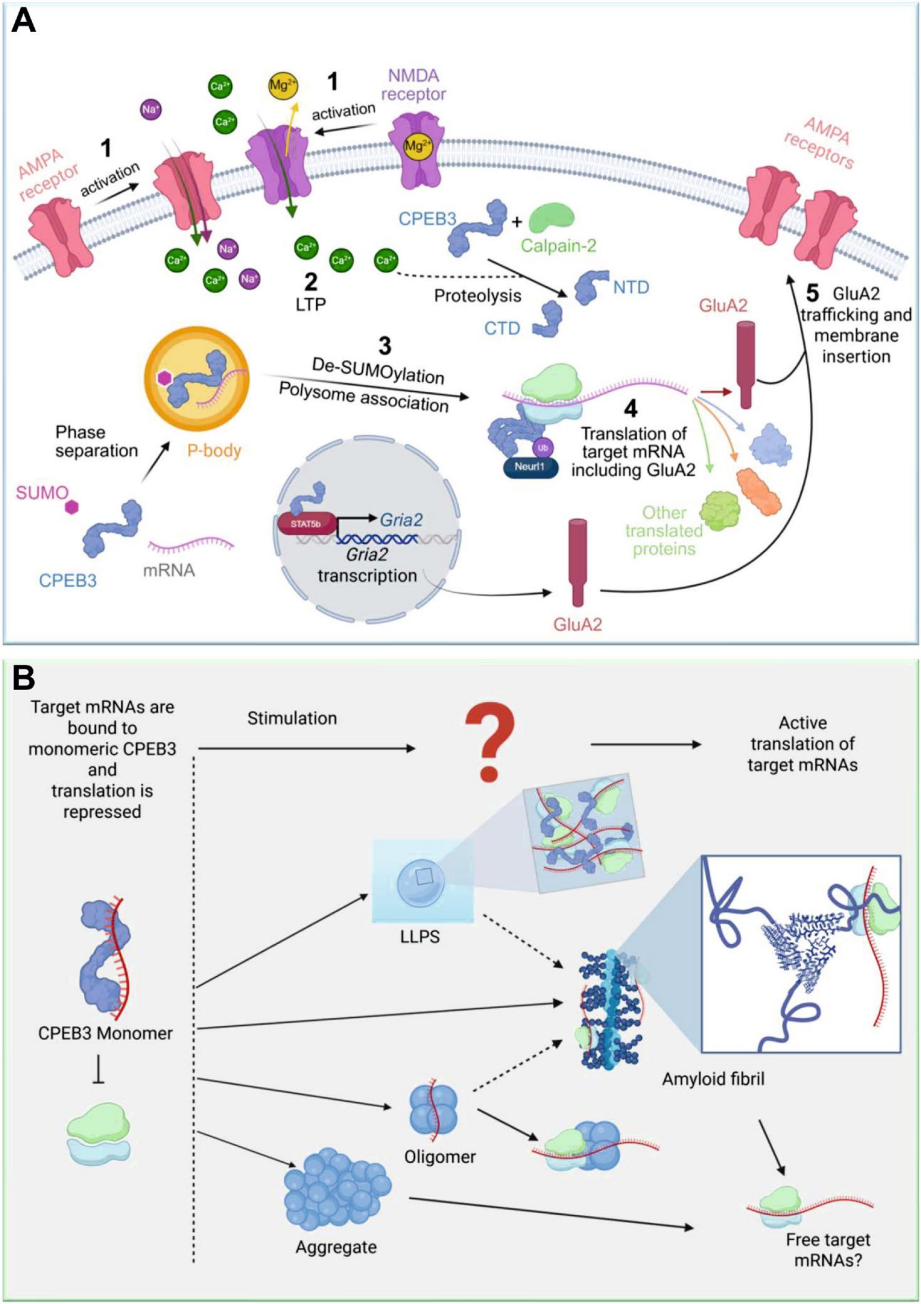
CPEB3 trafficking and regulation of AMPA receptors and possible modes of action of CPEB3 in memory. (A) SUMOylation of CPEB3 and its association with target mRNA can lead to phase separation and localisation to P bodies, where translation of target mRNAs is repressed. (1) Activation of AMPA and NMDA receptors leads to (2) LTP. Increased cellular Ca^2+^ concentration leads to activation of calpain‐2 and proteolysis of CPEB3 into its NTD and CTD halves. (3) LTP leads to deSUMOylation of CPEB3 and translocation out of P bodies. (4) Subsequent oligomerisation of CPEB3 and its mono‐ubiquitination by Neurl1 leads to CPEB3 association with polysomes and translation of target mRNAs, including GluA2. (5) This results in increased AMPA subunit trafficking to the cell surface and membrane insertion of functional AMPA receptors. In addition, CPEB3 is able to modulate GluA2 indirectly through STAT5b. Created in BioRender. Hicks, D. (2025) https://BioRender.com/tbnros4. (B) Possible structures into which CPEB3 (represented in blue) might self‐assemble. Oligomers (represented using blue beads), condensates (LLPS), aggregates (represented using blue beads) or amyloid‐like fibrils (represented using beads (light blue representing the core and dark blue representing the ‘fuzzy coat’) and also in detail using the PDB 6VPS model of Orb2 fibril structures (light blue) with additional ‘fuzzy coat’ regions (dark blue)). Each structure may actively facilitate translation of target mRNAs or result in their release. Which, if any, or all, of these is involved in long‐term formation of memory remains to be resolved and is a key question for future research. Created in BioRender. Aubrey, L. (2025) https://BioRender.com/nl6c5uk.

The synaptic changes associated with memory acquisition and stabilisation are mediated by long‐term potentiation (LTP) and long‐term depression (LTD) (de Ortega‐ San Luis and Ryan 2022; Merlo et al. 2024). LTP is associated with the activation of NMDA receptors and the subsequent increase of synaptic trafficking of AMPA receptors (ionotropic glutamate receptors) (Malinow 2003), and learning tasks can promote the recruitment of newly synthesised AMPA receptors to synapses (Matsuo et al. 2008), enhancing synaptic strength. Glutamate binding to post‐synaptic AMPA receptors leads to membrane depolarisation. This results in the removal of the magnesium ions blocking the NMDA receptors, allowing calcium influx into postsynaptic neurons which activates Ca^2+^/calmodulin‐dependent protein kinase II (CaMKII) (Figure 1). This results in the phosphorylation of GluA1 subunits in AMPA receptors and increases AMPA receptor trafficking to the synapse. In addition, CaMKII activity induces cytoskeletal rearrangements, which also contribute to structural remodelling of synapses. In late‐phase LTP, which supports long‐term memory persistence, the Ca^2+^ signalling activates the cAMP, PKA (Protein Kinase A) and MAPK (Mitogen‐Activated Protein Kinase) pathway. This cascade leads to the phosphorylation of the transcription factor, cAMP response element‐binding protein (CREB), promoting the expression of genes such as *Arc* and *Fos* that are critical for synaptic consolidation and memory persistence (de Ortega‐ San Luis and Ryan 2022; Pereyra and Medina 2021; de Leon‐Lopez et al. 2025).

The key difference between short‐ and long‐term memories is that the formation of stable memories, which need to be stored and retrieved, requires new gene expression. This involves a dual mechanism: the transcription of new mRNAs and the translation of pre‐existing, quiescent mRNAs localised at synapses. Cytosolic ribonucleoprotein (RNP) granules can sequester these mRNA in a translationally repressed state, preventing their translation until activated by specific stimuli. The reversible assembly of RNP granules is thought to be mediated by low complexity domains (LCDs) that are present in many RNA‐binding proteins (RBP) (Sudhakaran and Ramaswami 2017) and are known to be involved in liquid–liquid phase separation (LLPS) and condensate formation in many processes spanning virus assembly, ribosome biogenesis and neurodegeneration (Naskar et al. 2023) and, as postulated and reviewed here, potentially also in long‐term memory.

How then can long‐term memory (LTM) be maintained and persist far longer than the lifespan of the proteins initially synthesised to encode them? Francis Crick proposed that this problem could be solved by the existence of a ‘macromolecule which is relatively immune from molecular turnover’, although this idea was initially considered improbable (Crick 1984). Recent research has identified several plausible mechanisms that could contribute towards addressing this issue. Epigenetic modifications, such as DNA methylation and histone modifications, can stably alter gene expression patterns over long periods (Kupke and Oliveira 2025). Molecular scaffolds, such as the synaptic protein KIBRA, or the extracellular matrix that surrounds neurones known as perineuronal nets, have also been shown to stabilise memory‐related synaptic changes (Lev‐Ram et al. 2024). The upsurge of interest in prion biology in the late 1990s led to the suggestion of a prion‐based mechanism of long‐term memory storage, in which amyloid‐like fibrils, which inherently are highly stable and are able to self‐propagate, could be involved (Tompa and Friedrich 1998).

## 2 The CPEB Family of Proteins

### 2.1 Discovering CPEB

**FIGURE 2:**
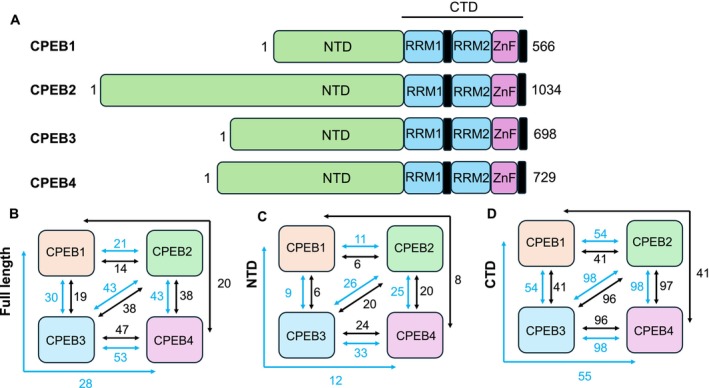
Domain organisation in CPEB family members. (A) Domain organisation in CPEBs 1–4, showing NTD and CTD with RNA Recognition Motifs (RRM) and zinc finger (ZnF) sub‐domains. (B–D) Percentage sequence identity (black) and similarity (blue) between CPEB family members. Similarity is defined as GAVLI/FYW/CM/ST/KRH/DENQ/P.

In 1983, Rosenthal and colleagues published a seminal paper linking mRNA adenylation and translational control (Rosenthal et al. 1983) (reviewed previously in Fernandez and Mendez 2025). A subsequent work in *Xenopus* identified a consensus uracil (U)‐rich sequence motif required for G10 mRNA polyadenylation. This motif was termed the cytoplasmic polyadenylation element (CPE), and cross‐linking experiments identified a corresponding binding protein, named CPEB (cytoplasmic polyadenylation element binding protein) (McGrew and Richter 1990). Further experiments with *Xenopus* B4 mRNA identified a CPEB of a different molecular weight, leading to the speculation of multiple CPEBs (Paris et al. 1991; Simon et al. 1992). This was followed by affinity purification of a 62 kDa CPEB protein that contains two RNA‐recognition motifs (RRMs) and a zinc‐finger motif in the C‐terminal region (Hake and Richter 1994; Hake et al. 1998; Merkel et al. 2013), in addition to an N‐terminal PEST domain (Reverte et al. 2001; Mendez et al. 2002). Cloning of the CPEB cDNA proved to be the gateway to further functional characterisation and established CPEB as a key regulator of cytoplasmic polyadenylation, activating dormant translationally inactive mRNA, especially in the context of development and the cell cycle (Stebbins‐Boaz et al. 1996). This discovery was later expanded by the identification of additional CPEB family members in vertebrates named CPEB2, CPEB3 and CPEB4 (Kurihara et al. 2003; Theis et al. 2003; Turimella et al. 2015) (Figure 2) which originate from a single ancestral gene (Vaglietti et al. 2025).

Consistent with its functional importance, CPEB is highly conserved across species, with *Aplysia californica* (California sea hare, ApCPEB) having two paralogues, one in each subfamily, *Drosophila* having two (Orb1 and Orb2) and vertebrates having four paralogues (CPEB1‐4, Figure 2) (Duran‐Arqué et al. 2022). Furthermore, it has been suggested that CPEB1 and CPEB2‐4 can be separated into different subfamilies (Rouhana et al. 2023).

The CPEB family of proteins has an unusual primary sequence composition and organisation: they contain relatively few lysine and glutamate residues (compared with the average across all proteins in the proteome), while serine and proline are over‐represented. In addition, the sequence is also asymmetrically organised, with proline and glutamine residues being concentrated in the N‐terminal domain (NTD) of CPEB2‐4 where they form long homo‐polymeric repeats, while these sequences are absent in CPEB1 (Figure 2). These regions have high scores for sequence simplicity and repetitiveness, and a high propensity to undergo LLPS, as predicted by tools such as FuzDrop and ParSe (Vaglietti et al. 2025).

Assessment of sequence similarity shows that there is a substantial difference between the sequence of CPEB1 and its family members, CPEB2 to4 (Figure 2B–D) (Rouhana et al. 2023; Mendez and Richter 2001). This suggests that CPEB1 and CPEB2 to4 may regulate distinct sets of RNA targets and operate through different mechanisms (Huang et al. 2006; Lee et al. 2016). Supporting this view, the regulatory pathways of CPEB1 and CPEB2 to4 are also different, with CPEB2 to4 being co‐regulated by miR‐26 and miR‐92 (Morgan et al. 2010; Winkler et al. 2023), while CPEB1 is regulated by miR‐22 (Fiumara et al. 2015). When over‐expressed individually as GFP fusions in mammalian cells, all four vertebrate CPEBs form foci, although with significant differences in number, sphericity and fluorescence intensity. CPEB1 and CPEB2 foci are larger than those of CPEB3 or CPEB4, and CPEB1 foci are less fluorescent and significantly more abundant than foci of the other CPEBs. CPEB3 foci, while fewer in number and lower in volume, display the highest fluorescence intensity of all CPEBs and also show the fastest recovery after photobleaching, suggesting dynamic molecular exchange. Overall, CPEB1 foci resemble RNP granules in which RNA is required for condensate formation, while CPEB2 to4 can undergo LLPS in the absence of RNA (Duran‐Arqué et al. 2022). In neurons, CPEB3 is present in RNP granules and P bodies, colocalising with Staufen‐2, Pumilio and FMRP. A mutant form of CPEB3 (S240‐242A) shows reduced co‐localisation with P bodies and inhibition to the translation of a reporter construct (Ford et al. 2023), linking a key role of CPEB3 both in RNP granule formation and translation control.

The high sequence variability in the NTD of CPEBs (Figure 2C) gives rise to disparate regulatory mechanisms of the different family members. For example, phosphorylation of CPEB1 at Ser174 by Aurora kinase A promotes the transition of the protein from a translational repressor to an activator (Sarkissian et al. 2004), with translational activation thereafter being controlled by degradation mediated by Cdc2, Cdk1 and Plk1 (Mendez et al. 2002; Setoyama et al. 2007). CPEB4, by contrast, is activated via phosphorylation at multiple sites in its NTD by ERK2 and Cdk1 (Guillen‐Boixet et al. 2016). CPEB3 regulation is modulated by post‐translational modifications such as mono‐ubiquitination and SUMOylation (Pavlopoulos et al. 2011; Drisaldi et al. 2015), the latter being linked to the localisation of the protein to cytoplasmic granules, including TDP‐43 (Verde et al. 2025; Keiten‐Schmitz et al. 2021; Yau et al. 2021). Furthermore, SUMOylation has been linked to synaptic function and memory formation more generally (Schorova and Martin 2016; Gustin et al. 2022).

Functionally, all CPEBs can recruit the CCR4‐NOT deadenylase to the CPEB–repressor complex. This recruitment is crucial for the regulation of mRNA stability and translation. However, only a fraction of transcripts regulated by CCR4‐NOT deadenylase is common to all four CPEBs, indicating specificity. While all CPEBs recognise the canonical cytoplasmic polyadenylation element (CPE, UUUUA_(1–2)_U), CPEB2‐4 also recognise the ‘G variant’ sequence (UUUUGU) (Fernandez and Mendez 2025; Duran‐Arqué et al. 2022) allowing a broader range of mRNA regulation. Together, these findings highlight the commonality in the role of CPEBs in translational control but highlight that the different family members have different mRNA targets, different mechanisms of activation and repression.

## 3 CPEBs In Neurons and Memory

Further detailed characterisation of CPEB throughout the 1990s, predominantly by Richter and coworkers, led to the discovery of CPEB as a regulator of local protein synthesis in neurons (Wu et al. 1998). This finding supported the broader concept that synaptic plasticity is underpinned by protein synthesis from dendritically localised mRNA, especially Ca^2+^‐calmodulin‐dependent protein kinase II (CaMKII) (Ouyang et al. 1997). In rodent brain, CPEB1 is localised at synapses, where it binds to the CPE in the 3'UTR of CaMKII mRNA, regulating its polyadenylation and translation (Wu et al. 1998). This regulatory mechanism is activated by NMDA receptor stimulation (Wells et al. 2001; Huang et al. 2002). Indeed, appending the CPE‐containing CaMKII 3'UTR to a GFP reporter mRNA enhances dendritic trafficking of the reporter (Huang et al. 2003). Furthermore, Ca^2+^ influx leads to CaMKII activation, which in turn phosphorylates CPEB1 at Thr171, modulating its activity, linking neuronal activation to CPEB1 activity in translational control (Atkins et al. 2004).

In vivo studies of CPEB1 knockout (KO) mice show no significant alterations in baseline behavioural characteristics. However, the KO mice exhibit impaired hippocampal memory extinction, as demonstrated in a swim‐maze task and a fear conditioning test (Berger‐Sweeney et al. 2006) and deficits in NMDA‐dependent long‐term potentiation (LTP) (Alarcon et al. 2004; Zearfoss et al. 2008). It has been suggested that the role of CPEB in NMDA‐dependent LTP is dependent on its phosphorylation by CaMKII (Atkins et al. 2005). Similar to CPEB1 KO mice, CPEB3 KO mice also show no changes in physical performance or behaviour in their home cage, but they have enhanced short‐term fear memory and long‐term spatial memory (Chao et al. 2013). Brain homogenates from these KO mice show elevated levels of NMDA receptor subunits (NR1, NR2A and NR2B), PSD‐95 and the AMPA receptor subunit GluA1 (Chao et al. 2013), consistent with previous findings that CPEB3 represses GluA2 translation (Huang et al. 2006) (Figure 1A). The consequent increase in NMDAR‐mediated Ca^2+^ influx leads to CaMKII hyperactivation and abnormal LTD responses (Huang et al. 2014). Recent findings also show that CPEB3 limits the nuclear translocation of the transcription factor, STAT3, reducing the expression of NMDAR subunits (You et al. 2025). CPEB3 has also been implicated in visual imprinting in the chick intermediate medial mesopallium (IMM), a brain region critical for linking visual input and learning. In this learning paradigm, aggregated CPEB3 was shown to be upregulated in the IMM after the training phase (Margvelani et al. 2018). Moreover, higher levels of aggregated CPEB3 showed a significant positive correlation with performance in the testing phase (Chitadze et al. 2023), suggesting a functional role of CPEB3 self‐assembly in memory consolidation. Similarly, increased detergent‐insoluble CPEB3 is seen after fear conditioning (Fioriti et al. 2015). Conditional knockout of CPEB3 in the mouse forebrain also leads to multiple memory impairments, including deficits in associative memory, spatial memory and memory maintenance (Fioriti et al. 2015). RNA immunoprecipitation has identified learning and memory as one of the top gene ontology categories for CPEB3 targets, including genes such as *Grm5*, *Kalrn* and *Nr3c1*. Furthermore, CPEB3 KO mice show increased hippocampal glucocorticoid receptor signalling and reduced expression of brain‐derived neurotrophic factor (BDNF), further supporting its role in cognitive performance and emotional regulation (Lu et al. 2021). In contrast to the effects of CPEB3, knockout of CPEB4 does not result in any memory deficits (Tsai et al. 2013). These findings highlight the distinct and diverse roles of CPEBs in regulating various aspects of memory, including synaptic plasticity, memory formation and maintenance.

Interestingly, there is a short, highly conserved intronic sequence in *CPEB3* that acts as a ribozyme, regulating its own expression. A substitution of U to C (rs11186856) in this sequence enhances self‐cleavage of CPEB3 by two‐fold, resulting in a concomitant decrease in CPEB3 protein expression (Salehi‐Ashtiani et al. 2006). Individuals with the homozygous *CC* genotype showed a deficit in semantic memory performance, while the heterozygote genotype had no effect (Vogler et al. 2009). Consequently, antisense oligonucleotide targeting of the *Cpeb3* ribozyme leads to increases in *Cpeb3* mRNA. This results in increased CPEB3 protein, alongside increases in GluA1, NR2B and PSD95 expression, leading to enhanced long‐term memory (Chen et al. 2024). Furthermore, this CPEB3 variant has also been recently linked to memory in the context of foreign language proficiency (Yerdenova et al. 2025). Collectively, this panoply of evidence points firmly to a role of CPEBs in memory formation, although the underlying cellular and molecular mechanisms remain complex and are not yet fully elucidated.

### 3.1 CPEBs in Invertebrates

Much valuable information about learning and memory has been garnered through the use of *Drosophila melanogaster* as a model system (Davis 2023). The *Drosophila* CPEB, Orb2, has been proposed to promote memory via its isoforms (Orb2A and Orb2B), but also through regulating genes involved in neuronal growth and synapse formation (Mastushita‐Sakai et al. 2010; Pai et al. 2013; Sanguanini and Cattaneo 2018; Stepien et al. 2016). Accordingly, deletion of the N‐terminal Gln‐rich region of Orb2 resulted in defects in long‐term, but not short‐term memories (Keleman et al. 2007), which is suggestive of a role for aggregation in memory‐related functions (Majumdar et al. 2012). This memory deficiency can be mitigated by expression of the lingerer protein (Lig), which can form a complex with Orb2 (Kimura et al. 2015). The function of Orb2 in memory may involve oligomerisation acting as a switch between its activating and repressive activities (Khan et al. 2015). Aggregation of Orb2A has been shown to be crucial for synapse‐specific activation of dormant mRNA and long‐term memory stabilisation (Majumdar et al. 2012; Khan et al. 2015; Krüttner et al. 2012). It has been reported that Orb2 oligomerisation is potentiated by Hsp40 family member Mrj and the DNA‐J family chaperone JJJ2 (Desai et al. 2024; Li et al. 2016). Subcellular localisation of Orb2 is dependent on its 3'UTR. Interestingly, 3'UTR deletion did not result in any change in soma expression of Orb2, but synaptic Orb2 was significantly reduced (Kozlov, Deev, et al. 2023; Kozlov, Tokmatcheva, et al. 2023).

Furthermore, Orb2A mRNA is present as a non‐protein coding unspliced transcript, where long‐term memory formation is potentiated by pasilla‐mediated Orb2A splicing to a protein coding transcript (Gill et al. 2017). It has been suggested that Orb2A is primarily involved in memory acquisition, with Orb2B required for consolidation (Krüttner et al. 2015). Orb2B may act as a conventional CPEB, involved in mRNA transport and regulation, but Orb2A is required for the formation of stable Orb2 complexes (Krüttner et al. 2012).

There have also been several interesting reports on the *Aplysia* CPEB (ApCPEB). This protein has been shown to have prion‐like properties (Si, Lindquist, and Kandel 2003; Si, Giustetto, et al. 2003), has high stability (Heinrich and Lindquist 2011) and can localise to RNP granules (Chae et al. 2010). A conformational switch in ApCPEB has been described, involving the conversation of soluble α‐helix‐rich oligomers to β‐sheet‐rich fibrils (Raveendra et al. 2013). Activation of 5‐HT receptors can downregulate miR‐22, leading to increased expression of ApCPEB and activation of target genes supporting long‐term facilitation (LTF, *Aplysia* equivalent of LTP) (Fiumara et al. 2015). However, this is dependent on local protein synthesis, as its inhibition blocks LTF (Miniaci et al. 2008). ApCPEB co‐operates with the granule protein Staufen to ensure correct localisation of syntaxin mRNA (Liu et al. 2006), a system which may also operate in mammalian cells (Ford et al. 2023).

### 3.2 CPEB In Neurodevelopmental Disorders and Neurogenesis

Adult neurogenesis is a key process underpinning learning and memory, primarily through the formation of new neurons from neural progenitor cells (NPCs) in the hippocampus (Chan et al. 2022). Recent work has revealed a key role for CPEB3 in modulating transcripts associated with neurogenesis, such as *Nav2, Lcn2 and Cyld* (Qu et al. 2020). CPEB3 regulates the alternative splicing of pre‐mRNA for these genes, thereby influencing the production of functionally distinct protein isoforms. *Nav2* (neuron navigator 2) is essential for axonal guidance and brain development (Accogli et al. 2023) and has been identified as a genetic risk factor for Alzheimer's disease (Yan et al. 2015; Wang et al. 2017). *Lcn2*, which encodes lipocalin‐2, contributes to neurogenesis, stem cell proliferation and neuroinflammation and has been linked to performance in spatial memory tasks (Ferreira et al. 2013; Ferreira et al. 2018). *Cyld* encodes a lysine 63 deubiquitinase involved in synaptic signalling and fear memory formation (Li et al. 2025). Mutations of Cyld have been linked to memory loss in frontotemporal dementia (Tábuas‐Pereira et al. 2020). Finally, CPEB3 regulates FosB, a transcription factor that is critically involved in hippocampal neurogenesis (Manning et al. 2019; Drisaldi et al. 2020), further highlighting the importance of CPEB3 in the molecular regulation of adult brain plasticity. Together, the findings highlight the importance of translational control of neuronal function and a key role of CPEBs in this activity.

CPEB3 has been implicated in the neurodevelopmental disorder Fragile X Syndrome (FXS). Here CPEB3 is reported to act as a downstream effector of Fragile X mental retardation protein (FMRP) which is deficient in individuals with FXS. In *Fmr1* KO model mice (*Fmr1* encodes FMRP), CPEB3 binds the *Gria2* promoter and enhances STAT5b‐driven transcription, resulting in elevated GluA2 expression and altered AMPAR function (Hwang et al. 2022). The nuclear import of CPEB3 has also been shown to be linked to NMDAR activation (Chao et al. 2012). The finding that the *Fmr1* KO phenotype can be ameliorated by concomitant knockout of CPEB1 supports the notion that these different CPEB family members have distinct roles in controlling translation in neurons (Udagawa et al. 2013).

Recent work has also linked CPEB function to Down's syndrome and autism spectrum disorder (ASD), both of which can be associated with learning and memory deficits (Casañas et al. 2019; Montesinos 2017). This is mediated, at least in part, via the modulation of Down's syndrome cell adhesion molecule (DSCAM), which plays a critical role in neural development and synaptic connectivity (Montesinos 2017; Jain and Welshhans 2016). DSCAM has been shown to associate with CPEB1 in the dendrites of the hippocampus, regulating local translation of DSCAM upon neuronal activation (Alves‐Sampaio et al. 2010). Dysregulation of DSCAM expression has not only been linked to the cognitive deficits observed in Down's syndrome (Emili et al. 2024) but also to autism‐like behaviours and impairments in spatial memory in animal models (Chen et al. 2022; Neff et al. 2024). These findings suggest that CPEB‐mediated control of DSCAM expression may represent a convergent molecular mechanism underlying shared cognitive phenotypes across these disorders.

It has been suggested that addictive behaviour is an example of perturbed learning and memory pathways (Thomas et al. 2008; Ding et al. 2021). Interestingly, cocaine administration in mice causes significant increases in striatal CPEB1 and CPEB3 expression, while mice deficient in CPEB1 and CPEB3 showed reduced addiction behaviours, again linking CPEBs to learning and memory. Cocaine administration also caused significant increases in the transcription factor ΔFosB, which is closely associated with addiction. This increase was not seen in CPEB1/3 knockout mice, suggesting that CPEB1/3 may regulate translation of FosB and its splice variant ΔFosB (Drisaldi et al. 2020). In addition to well‐described roles in addiction, FosB is also involved in spatial memory (Solecki et al. 2008; Lamothe‐Molina et al. 2022) and learning (Manning et al. 2019), and targeting ΔFosB signalling has been shown to restore memory in a model of Alzheimer's disease (Corbett et al. 2017). In addition, rodents with sub‐chronic exposure to aluminium were shown to cause learning and memory deficits, mediated by miR‐353‐5p repression of CPEB3 (Ji et al. 2024). Similarly, exposure to prenatal diesel exhaust particulates resulted in reduced CPEB3 expression, alterations in NMDAR subunit expression and defects in hippocampal learning and memory (Yu et al. 2024). Hence, exposure to drugs and other potentially toxic compounds that are associated with memory deficits is united by a role of CPEB in translational control.

Overall, these data highlight the central role of CPEBs in gene regulatory hubs that govern brain development and memory. Further understanding the function of CPEBs could provide further insights on both neurodevelopmental and neurodegenerative disorders. While these novel findings may pave the way for novel therapeutic strategies, it is unlikely that direct targeting of CPEB3 is a viable therapeutic option given its links to cancer, including neuroblastoma (Yang et al. 2021).

## 4 CPEB Structure

### 4.1 Domain Organisation

**FIGURE 3:**
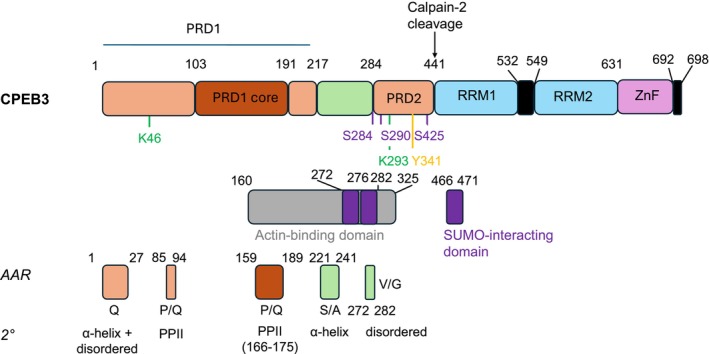
Domain organisation in human CPEB3. Top: The main functional domains of CPEB3. Middle: Residues 160–325 (the Actin binding domain). Lower: Regions with repetitive sequences (AAR (amino acid repeat) regions) and those able to form transient secondary structural elements of different types (2°).

AlphaFold predicted structures of all CPEB paralogues reveal a predominantly disordered NTD with some structured segments interspersed throughout (Vaglietti et al. 2025). NMR analysis of the NTD of CPEB3 using^13^C labelled protein confirmed these models, showing the disordered nature of the NTD, which lacks secondary structure across the 426‐residue region (Figure 3). However, transient alpha‐helix formation, involving residues 1–10, 202–210, 222–234 and 238–246, is observed, along with partially populated polyproline II helices (PPII) at residues 86–93 and 166–175, highlighting the potential for these regions to form structured elements (Ramírez de Mingo et al. 2022). Two prion‐like domains (PrLD) have been identified (PRD1: residues 1–217 and PRD2 residues 284–449 in mouse CPEB3). When expressed and purified, PRD1 was shown to be capable of forming amyloid‐like fibrils (Flores et al. 2025; Reselammal et al. 2021), with the amyloid core region involving residues 101–194, as identified by hydrogen‐deuterium exchange (HDX‐NMR) and limited proteolysis (Reselammal et al. 2021). This supports the view that self‐assembly into amyloid‐like fibrils potentially could be involved in the function of CPEB3, suggesting that CPEBs might belong to the growing class of functional amyloids (Kozlov et al. 2021).

Across different animal classes, the NTD of CPEB3 homologues is variable among different sequences, with similarity to human CPEB3 ranging from 94% in dolphin to 49% in zebrafish (Table 1). In contrast, the C‐terminal domain (CTD) (residues 442–698 in human CPEB3; Figure 3) is highly conserved, with sequence similarity to human CPEB3 ranging from 99% in fish to 100% in all other classes (Table 1). This suggests a strong evolutionary pressure to conserve the RNA‐binding CTD, a constraint not observed in the more variable NTD.

**TABLE 1:** Sequence similarity of CPEB3 between animal classes.

	Full length	NTD (1–441)	CTD (442–698)
Human	100	100	100
Mouse	95	92	100
Dolphin	96	94	100
Frog	75	62	100
Tortoise	85	76	100
Alligator	85	78	100
Chicken	85	77	100
Python	83	74	100
Lungfish	76	65	99
Zebrafish	66	49	99
Shark	76	63	99

*Note:* Percentages represent sequence similarity to the human sequence of CPEB3 defined as GAVLI/FYW/CM/ST/KRH/DENQ/P. The border between NTD and CTD is defined by RRM1, independently of residue number; 1–441 and 442–698 refer to the human sequence only.

Within the NTD of human CPEB3, residues Q164–T325 form an actin‐binding domain (ABD). This contains a chameleon sequence (R192‐K220), which can adopt two different secondary structure conformations: α‐helix or β‐hairpin. Binding of F‐actin to the CPEB3 ABD triggers aggregation by promoting β‐hairpin formation in this chameleon sequence (Gu et al. 2020). In addition, SUMOylation of CPEB3, potentially at K220, potentially could inhibit the ABD–F–actin interaction, modulating the conformational preferences of this region, affecting CPEB function (Drisaldi et al. 2015; Gu et al. 2020; Gu et al. 2022).

PrLDs, such as those found in CPEB3, are commonly associated with RNP assemblies (Boncella et al. 2020), many of which are structurally defined but functionally ambiguous (Ripin and Parker 2023). In cultured mouse neurons, CPEB3 has been shown to colocalise with Staufen‐2 and FMRP in neuronal RNP granules, both of which are associated with memory formation (Sudhakaran and Ramaswami 2017; Ford et al. 2023). Several other RBPs, including Gadd45α and the ELAV family, have also been implicated in memory formation (Di Liegro et al. 2024; Aparisi Rey et al. 2019; Mirisis and Carew 2019). These findings suggest that self‐assembly of CPEBs into higher‐order assemblies may be important in its function, as we discuss below.

### 4.2 Functional Amyloid and CPEB3

Amyloid fibrils are composed of protofilaments which are linear stacks of monomeric protein subunits arranged in β‐sheet layers spaced approximately 4.7–4.9 Å apart (Taylor and Staniforth 2022; Benson et al. 2018). Although pathological amyloid assemblies have been linked to neurodegenerative diseases, such as Alzheimer's disease and Parkinson's disease (Gkanatsiou et al. 2021), several examples of functional amyloids exist. These can be reversible, are typically stimulus‐dependent and serve disparate physiological functions. Examples include amyloids in bacteria (CsgA and FapC, involved in biofilm formation), in archaea (MspA, wherein amyloid forms the protective outer sheath) (Otzen et al. 2021) and in humans (storage of peptide hormones (Maji et al. 2009); promoting cell migration (fibronectin) (Bascetin et al. 2017), synthesis of melanin in melanosomes (Pmel17) (Berson et al. 2003) and in cell death caused by necroptosis (RIPK1 and RIPK3)) (Buchanan et al. 2023).

*Drosophila* Orb2 has been shown to form amyloid fibrils in which the fibril core comprises just 31 residues from its 704 amino acid sequence. Structural solution of these fibrils, extracted directly from the brains of *Drosophila*, using cryogenic electron microscopy (cryoEM) showed that the amyloid fibril core adopts a cross‐β structure stabilised by interdigitated glutamine residues that form a hydrophilic hairpin (Hervas et al. 2020) (Figure 1B). The fibril core is separated from the RNA‐binding domains by long, flexible regions rich in glycine and serine residues, offering high conformational flexibility to these regions in a fuzzy ‘halo’ that surrounds the fibril core (see image in Figure 1B) (Hervas et al. 2020). Immunogold labelling has demonstrated a potential functional switch from a translation repression complex in the monomeric state to a translation activation complex in the fibrillar state, upon which target mRNA remains bound (Hervas et al. 2020), strongly suggesting that, at least for Orb2, protein self‐assembly is involved in its functional role in translation control (Figure 1B).

In *Drosophila melanogaster* , two Orb2 proteins appear to work in tandem, with Orb2B being far more highly expressed and also more resistant to aggregation. Orb2A and Orb2B have common glutamine‐rich and RNA‐binding domains, but differ in their N‐terminal sequences: Orb2A has a short, unique sequence before its Gln/His‐rich region, while Orb2B has a longer, serine‐rich region at its N‐terminus (Cervantes et al. 2016). Orb2A is thought to be essential for the formation of Orb2 fibrils; despite 97.5% of the total monomeric subunits in ex vivo Orb2 fibrils being Orb2B (Hervas et al. 2020), fibril assembly does not occur in the absence of Orb2A. Hence, Orb2A is required to initiate Orb2B fibril formation (Majumdar et al. 2012). In addition, calmodulin binding to Orb2A can inhibit fibril formation (Soria et al. 2022). The Orb2A N‐terminal domain is 153 residues shorter than Orb2B and in isolation, the N‐terminal 88 residues of Orb2A rapidly assemble into amyloid fibrils, albeit with a different structure and including different residues in the ordered core when compared with the ex vivo fibril structure (Cervantes et al. 2016). The N‐terminal 20 residues on Orb2A have also been demonstrated to bind lipid bilayers, perhaps regulating Orb2A (and therefore all Orb2) fibril assembly (Soria et al. 2017). In vertebrates, CPEB3 appears to work in isolation, and so the mechanism for its fibril assembly differs significantly from Orb2.

Proteins enriched in glutamine (or asparagine) residues have been shown to form reversible amyloid‐like inclusions in yeast that are required for the inheritance of phenotypic features, and hence to have prion‐like capabilities (Tuite 2000). Aligned with these findings, a Gln/Asp‐rich region in ApCPEB was also found to enable the formation of insoluble, aggregated multimers, which has been implied to support long term synaptic changes (Si, Lindquist, and Kandel 2003; Si, Giustetto, et al. 2003; Miniaci et al. 2008; Si et al. 2010; Liu and Schwartz 2003; Rahn et al. 2013). This self‐sustaining biochemical reaction and continuous macromolecular synthesis would offer a potential solution to the paradox of how long‐lasting synaptic modifications can persist despite the continual turnover of synaptic proteins (Crick 1984; Kandel 2001): just the kind of protein that Crick suggested would be required for memory formation (Crick 1984).

Based on the sequence similarity to these Gln/Asp‐rich proteins in its NTD, CPEB3 has been proposed to form functional amyloids, though direct in vivo evidence remains limited. In vitro, PRD1 can form fibrils as shown by negative stain EM. These fibrils bind Congo Red and Thioflavin T and exhibit β‐sheet rich circular dichroism spectra, classic features of amyloid (Flores et al. 2025; Reselammal et al. 2021; Benson et al. 2018) Furthermore, detergent‐insoluble CPEB3 has been isolated from chick brain and cultured neurons (Chitadze et al. 2023; Fioriti et al. 2015). However, amyloid‐like CPEB3 assemblies have not been observed definitively in vivo or ex vivo following physiological stimuli. With respect to their primary sequences, CPEB2 and CPEB3 have the lowest sequence complexity and highest repetitiveness among the vertebrate paralogues, features known to facilitate both LLPS and amyloid fibril formation (Vaglietti et al. 2025). Hence, despite much excitement and interest, the functional role of protein self‐assembly in vertebrate CPEB function remains speculative due to the lack of structural information on the insoluble aggregates in a physiological context (Huang et al. 2023) (Figure 1B).

### 4.3 Glutamine‐Rich Regions and a Role of Proline in CPEB3

Protein sequences with high glutamine content have long been linked to aggregation propensity (Shattuck et al. 2017). These regions can be divided into two broad categories: those that contain poly‐glutamine tracts, such as huntingtin, members of the ataxin family, TATA‐box‐binding protein and the androgen receptor (Scherzinger et al. 1997). Generally, the poly‐glutamine tract in these proteins is expanded in length in disease. For example, in huntingtin (associated with Huntington's disease), there are 6–35 glutamine residues in the poly‐Gln tract in normal cells, and this is expanded to 36–121 repeats in disease (Bonsor et al. 2024). The second category includes proteins that contain Gln‐rich regions in their sequence. CPEB3 belongs to this second category of proteins. Human CPEB3 contains a Gln‐rich region between residues Gln13 and Gln27, which contains 10 glutamine residues in total, but no more than four consecutive glutamines (Ford et al. 2019).

Interestingly, several of these proteins have proline‐rich regions adjacent to the poly‐Gln tract (e.g., ataxin 2, ataxin 7 androgen receptor, huntingtin). In the case of huntingtin, expansion of the proline‐rich region has been shown to increase protein solubility and mitigate the toxicity associated with poly‐Gln tract expansion, suggestive of a role of the proline‐rich region in protecting the proteins from aggregation (Pigazzini et al. 2021). Notably, CPEB3 also contains Gln‐rich regions interspersed with proline residues (Figure 3) (Ford et al. 2019). This pattern of proline‐rich regions flanking Gln‐rich segments is also observed in other RNA‐binding proteins such as TIA‐1 (T‐cell Intracellular Antigen 1), TIAR (T‐cell intracellular antigen 1‐related protein), FUS (Fused in Sarcoma protein) and SFPQ (Splicing Factor Proline/Glutamine rich protein), all of which are associated with the formation of dynamic cytosolic granules (Gilks et al. 2004; Huang et al. 2024; Udan and Baloh 2011). Mutation of the proline‐rich region in TIA‐1 has been shown to promote fibril formation and reduce granule disassembly (Ding et al. 2021), further supporting a functional role for the proline‐rich segments in modulating aggregation. In CPEB3, PPII helices that form in these protein‐rich segments may contribute to the formation of insoluble CPEB3 assemblies (Ramírez de Mingo et al. 2022). The proline content and poly(proline) length in CPEB3 have increased over evolutionary time, particularly in primates compared to cartilaginous fish *Chondrichthyes* (Vaglietti et al. 2025), suggestive perhaps of specific roles of the balance of proline‐rich and glutamine‐rich sequences in different organisms.

Overall, therefore, it is evident that the conserved and unusual patterning of the amino acid sequence in CPEBs is of functional significance and that their sequences may facilitate protein self‐assembly into aggregates, reversible condensates and/or stable amyloid fibrils, possibly involving binding of RNA via their RRMs. However, there is still much to learn about whether and precisely how these sequence features and their biophysical properties contribute to memory formation.

## 5 Progress and Extant Questions

The progress in the field of CPEB proteins, particularly CPEB3, in learning and memory can be assessed by the robustness and quality of the data addressing three key questions:

- Does CPEB3 have a role in human memory or related functions?
- What are the functional consequences of CPEB3 dysfunction?
- What are the molecular mechanisms underpinning CPEB3 function?

Two papers have directly addressed the role of CPEB3 in human memory, by Vogler et al. (2009) and Yerdenova et al. (2025). These reports assessed the correlation of the rs11186856 SNP (intronic CPEB3 variant) with performance in an episodic memory task or foreign language proficiency, respectively. This SNP is located in the CPEB3 ribozyme and is associated with enhanced self‐cleavage. The data from Vogler et al. (2009) report a strong correlation between CPEB3 and outcome in a memory assessment, but the population size was modest (total cohort 333 individuals) and causation cannot be inferred. Yerdenova et al. (2025) reported CPEB3 as part of a cohort of six other genes and foreign language proficiency was self‐reported. The only other *CPEB3* SNP (rs855708, intronic) linked with brain function is significantly associated with a panel of psychiatric disorders. However, it is of note that this was a genome‐wide association study; hence, *CPEB3* was one of many genes reported (Ding et al. 2023).

The only direct investigation of CPEB expression in human brain samples showed significant increases in CPEB1 and decreases in CPEB4 in Huntington's disease brains, but with no significant changes in CPEB2 or CPEB3 (Picó et al. 2021). In addition, risk genes for ASD contain CPEs and are bound by CPEB4, which is mis‐spliced in ASD, leading to risk gene deadenylation (Parras et al. 2018).

It may be possible to make some inferences on the involvement of CPEB3 in memory dysfunction via analysis of regulatory microRNA. It has been reported that CPEB3 is regulated by miR‐26, miR‐92 (Morgan et al. 2010), miR‐351 (Ji et al. 2024) and miR‐21 (Wang et al. 2021) in neuronal models and miR‐20 in non‐neuronal cells (Li et al. 2021). Furthermore, miR‐92 is upregulated in neuronal extracellular vesicles from individuals diagnosed with frontotemporal dementia (Manzini et al. 2025) and in Alzheimer's disease (AD) brain (Gugliandolo et al. 2020) and plasma samples (Siedlecki‐Wullich et al. 2019). Interestingly, miR‐92‐mediated repression of CPEB3 was beneficial in a mouse model of experimental autoimmune encephalomyelitis (Winkler et al. 2023). miR‐20 and miR‐26 are also increased in AD brain and blood samples, respectively (Wang et al. 2022; Tuna et al. 2025), but regulation of CPEB3 by miR‐20 has only been shown in cultured hepatocellular carcinoma cells (Li et al. 2021).

Overall, robust evidence for the role of CPEBs in human memory is sparse, based predominantly on a single study (Vogler et al. 2009), future work is needed. Data supporting the role of CPEBs in diseases of memory dysfunction are also still speculative at the current time, with evidence predominantly based on changes in regulatory microRNAs. The complexity here is illustrated by the reported increase in miR‐26 in AD (Tuna et al. 2025), which may be expected to repress *CPEB3* but also targets *BDNF*, which has a central role in AD (Phillips et al. 1991). Therefore, inferring a causal role for CPEB3 from these data is challenging.

In terms of mechanism, supporting data can be separated into structural and functional. Structurally, although recombinant CPEB3 PRD1 has been shown to form fibrils and the structure solved to high resolution using cryo‐EM (Flores et al. 2025; Reselammal et al. 2021), this has not yet been shown to be the case for the full‐length protein. Similarly, although Orb2 fibrils have been extracted from *Drosophila* brain and their structure solved to near atomic resolution (Hervas et al. 2020), this has not been reported for any vertebrate CPEBs. In addition, there has been no demonstration of CPEB3 amyloid formation in cultured cells as determined by either fluorescent amyloid‐specific dyes or by their extraction and structural characterisation. The notion of CPEB3 inclusion formation in vitro and in vivo rests on the formation of microscopic puncta or detergent‐insoluble species (Chitadze et al. 2023; Fioriti et al. 2015). So, while it is possible that CPEB3 forms amyloid fibrils, this remains to be demonstrated.

Given that CPEB3 contains canonical RNA‐recognition motifs (Tsuda et al. 2014) and the presence of similar domains in a range of other RNA‐binding proteins (Chan et al. 2022), it is highly likely that CPEB3 functions as an RNA‐binding protein in human neurons. Furthermore, RNA immunoprecipitation experiments have demonstrated CPEB3 binding to a range of targets in cultured mouse cells and mouse brain (Chao et al. 2013; Qu et al. 2020). However, transcriptomic analysis of this kind has yet to be performed in human cells or tissue; hence, the RNA targets of CPEB3 in humans remain unclear.

The existence of temporally regulated RNA granules, including P bodies, is now well‐established, and these granules can be purified (Riggs et al. 2020; Munier Godebert et al. 2025). RBPs have been linked to these granules, including as part of a cellular stress response (Molliex et al. 2015; Riback et al. 2017). The question, therefore, is the extent to which it has been demonstrated that CPEB3 can be recruited to P bodies or other granules. CPEB3 has been shown to colocalise with P‐body (Ford et al. 2019) and neuronal RNP markers (Ford et al. 2023), albeit using overexpression in mouse or non‐neuronal cells. So, while there is some good evidence for CPEB3 localisation to RNA granules, this remains to be demonstrated for endogenous CPEB3 in human neuronal cells. Similarly, these caveats also apply to the regulation of CPEB3 by SUMOylation and the ability of CPEB3 to regulate glutamate receptor subunits (Huang et al. 2006; Drisaldi et al. 2015; Stephan et al. 2015).

Delineating the precise role of CPEB3 in memory is challenging. Initially, it was reported that CPEB3 knockout mice displayed enhancement of both short‐term fear conditioning and long‐term spatial memory (Chao et al. 2013; Lu et al. 2021). Similarly, inhibition of miR‐92 leads to CPEB3 activation in mouse hippocampus, which impaired fear conditioning (Vetere et al. 2014). Yet, CPEB3 knockout mice were also shown to have impaired hippocampal LTP and spatial memory (Fioriti et al. 2015). Conversely, aluminium‐induced long‐term spatial memory deficits in rats were alleviated by miR‐351 inhibition and recovery of CPEB3 expression (Ji et al. 2024). In line with this, inhibition of the *Cpeb3* ribozyme increased CPEB3 expression and improved object location memory (Chen et al. 2024). The ability of CPEB3 to form detergent‐insoluble species has also been linked to learning processes, specifically filial imprinting in chicks and spatial memory in mice (Margvelani et al. 2018; Chitadze et al. 2023; Fioriti et al. 2015). It is likely that these conflicting data on the precise role of CPEB3 in memory derive from two potential opposing functions of CPEB3. Chao et al. (2013) reported that CPEB3 was acting to repress key transcripts encoding GluA1, PSD95 and NMDA receptor subunits, which may have been due to sequestration of these transcripts in RNA granules (Ford et al. 2019). However, Chen et al. (2024) reported the opposite effect, insofar as CPEB3 enhanced expression of these proteins, ascribing this to increased polyadenylation of the relevant mRNA transcripts.

It is likely that CPEB3 is able to repress mRNA translation via sequestration and activate via polyadenylation, based on unclear, but potentially highly dynamic, regulatory mechanisms. Published data support a role for CPEB3 in memory processes, but the precise mechanism for any such roles remains unclear. Future analysis of human tissue and biofluids alongside advanced human cell models, such as neurons derived from induced pluripotent stem cells, will be needed to further our understanding.

Of course, the most significant overarching question is how these data integrate with Crick's prediction from over 40 years ago. Might CPEB3 represent a ‘macromolecule which is relatively immune from molecular turnover’ (Crick 1984)? CPEB3 certainly has a primary sequence consistent with a protein capable of forming aggregated assemblies and certain isolated domains can form fibrils in a cell‐free system (Flores et al. 2025). Learning‐related tasks can induce the formation of detergent‐insoluble CPEB3 species (Fioriti et al. 2015). However, the cellular lifespan of these species is unclear, i.e., the extent to which they are relatively immune from molecular turnover or are able to template their structure onto newly synthesised CPEB monomers via fibril seeding activity. A pulse chase experiment could provide such information, but the ability to generate insoluble aggregates of endogenous CPEB3 in cultured cells has not yet been demonstrated. Also unclear is how the process of aggregation and amyloid formation regulates the apparent switch between target transcript polyadenylation or sequestration, although there are some interesting findings in *Drosophila* as to how this might be achieved (Khan et al. 2015).

## 6 Concluding Remarks

Memory encoding and retrieval remain an enigmatic phenomenon. This review presents key evidence supporting the role of CPEBs in maintaining long‐term memory. There is a wealth of data now that supports a role of CPEB3 in regulating the translation of dormant mRNAs at synapses, including those encoding AMPA and NMDA receptor subunits. This regulation enhances synaptic trafficking and modulates synaptic strength, both of which are essential for long‐term memory consolidation. Structurally, CPEB3 is well suited for this function. Its disordered NTD, enriched in glutamine and proline residues, promotes reversible aggregation and phase separation. These properties enable its localisation to ribonucleoprotein granules and may support the formation of functional amyloids. The conserved CTD contains RNA recognition motifs that are critical for binding target mRNAs. Together, these domains allow CPEB3 to act as a molecular switch, linking transient synaptic signals to sustained protein synthesis.

Despite these advances, many questions remain. It is not yet clear whether CPEB3 aggregation depends solely on NMDA receptor signalling, or if other stimuli, such as cellular stress, can also trigger this process. The role of SUMOylation in relieving translational repression, the formation and reversibility of amyloid‐like structures in vivo and the extent of CPEB3 regulation beyond glutamatergic pathways are all active areas of investigation. Additionally, the functional significance of proteolytic cleavage remains to be determined. A more detailed understanding of the individual sequence regions and any potential binding partners would be of great interest, and addressing such key extant questions will drive this exciting field forward in the future, so that the enigmatic question of how long‐term memories are made and retrieved at a molecular level may soon be better understood.

## Author Contributions

**David A. Hicks:** conceptualization, writing – original draft, visualization, writing – review and editing. **Liam D. Aubrey:** writing – original draft, visualization, writing – review and editing. **Jessica C. F. Kwok:** writing – review and editing. **Sheena E. Radford:** writing – review and editing.

## Conflicts of Interest

The authors declare no conflicts of interest.

## Peer Review

The peer review history for this article is available at https://www.webofscience.com/api/gateway/wos/peer‐review/10.1111/jnc.70226.

## Data Availability

Data sharing is not applicable to this article as no new data were created or analyzed in this study.
